# Overexpression of the PAP1 Transcription Factor Reveals a Complex Regulation of Flavonoid and Phenylpropanoid Metabolism in *Nicotiana tabacum* Plants Attacked by *Spodoptera litura*

**DOI:** 10.1371/journal.pone.0108849

**Published:** 2014-09-30

**Authors:** Tomoko Mitsunami, Masahiro Nishihara, Ivan Galis, Kabir Md Alamgir, Yuko Hojo, Kohei Fujita, Nobuhiro Sasaki, Keichiro Nemoto, Tatsuya Sawasaki, Gen-ichiro Arimura

**Affiliations:** 1 Department of Biological Science & Technology, Faculty of Industrial Science & Technology, Tokyo University of Science, Tokyo, Japan; 2 Iwate Biotechnology Research Center, Kitakami, Japan; 3 Institute of Plant Science and Resources, Okayama University, Kurashiki, Japan; 4 Proteo-Science Center, Ehime University, Matsuyama, Japan; Institute of Genetics and Developmental Biology, Chinese Academy of Sciences, China

## Abstract

Anthocyanin pigments and associated flavonoids have demonstrated antioxidant properties and benefits for human health. Consequently, current plant bioengineers have focused on how to modify flavonoid metabolism in plants. Most of that research, however, does not consider the role of natural biotic stresses (e.g., herbivore attack). To understand the influence of herbivore attack on the metabolic engineering of flavonoids, we examined tobacco plants overexpressing the *Arabidopsis PAP1* gene (encoding an MYB transcription factor), which accumulated anthocyanin pigments and other flavonoids/phenylpropanoids. In comparison to wild-type and control plants, transgenic plants exhibited greater resistance to *Spodoptera litura*. Moreover, herbivory suppressed the PAP1-induced increase of transcripts of flavonoid/phenylpropanoid biosynthetic genes (e.g., *F3H*) and the subsequent accumulation of these genes' metabolites, despite the unaltered *PAP1* mRNA levels after herbivory. The instances of down-regulation were independent of the signaling pathways mediated by defense-related jasmonates but were relevant to the levels of PAP1-induced and herbivory-suppressed transcription factors, An1a and An1b. Although initially *F3H* transcripts were suppressed by herbivory, after the *S. litura* feeding was interrupted, *F3H* transcripts increased. We hypothesize that in transgenic plants responding to herbivory, there is a complex mechanism regulating enriched flavonoid/phenylpropanoid compounds, via biotic stress signals.

## Introduction

The production of specialized metabolites in plants has attracted great interest as a result of the successful development of mechanisms that enable metabolic enzymes to be produced [1]–[3]. To synchronously activate metabolic genes, plants use a large variety of transcription factors. These factors may control multiple genes or orchestrate entire metabolic pathways or their specific branches. PRODUCTION OF ANTHOCYANIN PIGMENT1 (PAP1) is a typical R2R3 MYB-type transcription factor in *Arabidopsis thaliana* that is able to ectopically activate the entire array of genes involved in the biosynthesis of phenolic acids and flavonoid compounds (including anthocyanins) in the flavonoid biosynthetic pathway in several plant species, including tobacco, salvia, petunia and rose [4]–[8]. The overexpression of *Arabidopsis PAP1* in transgenic tobacco plants under the control of a constitutive promoter led to the induction of anthocyanins in the leaves, flowers, stems and roots; the deep red or purple appearance of the pants parts was evidence [5]. Moreover, *PAP1*-overexpressing petunia flowers have been shown to increase not only levels of anthocyanins but also emissions of floral volatiles (benzenoids) [7]. Gene expression profiling and measurements of the levels of pathway intermediates suggest that both increased metabolic flux and the transcriptional activation of scent and color genes underlie the enhancement of petunia flower color and scent production [7]. The coordinated regulation of metabolic steps within or between pathways involved in vital plant functions – for instance, pathways involved in floral display determining plant-pollinator interactions – provides plants with a clear advantage. Indeed, bees and humans can discriminate between the floral scents of *PAP1*-overexpressing and control rose flowers [6]. Therefore, PAP1 overexpression may find applications in horticulture and human health.

Manipulating flavonoid metabolism, especially in response to biotic or abiotic stresses, may have unpredicted side effects, as these compounds belong to the largest group of specialized metabolites produced by plants [9]. Flavonoids serve as essential components of a number of structural polymers that provide protection; lignin, for instance, due to its antioxidant and free radical scavenging properties, protects plants from ultraviolet light and defends plants against herbivores and pathogens [10]. Anthocyanin accumulation in *Arabidopsis* is enhanced in response to a number of environmental stress factors, such as cold, drought, pathogen attack and nutrient depletion [11]. Moreover, it appears that transgenic tobacco expresses structural genes and regulatory genes involved in flavonoid biosynthesis, and these genes defend the plant against herbivores [12], [13]. Therefore, *PAP1*-overexpressing plants that typically accumulate large amounts of flavonoids might possess increased plant resistance to biotic stresses, including herbivores, but to date no practical evidence of this prediction has been provided. Whether the *PAP1* transgene interferes with endogenous TFs and processes involved in plants' defense and development has not been extensively investigated.

A separate branch of flavonoid catabolism controlled by other MYB transcription factors (e.g., MYBJS1) was previously shown to control the accumulation of anti-herbivore compounds, phenolamides (PAs), in *Nicotiana* species [14]–[17]. In addition, CoA-activated phenolic acids with polyamines in PA production is controlled by the MYBJS1 homologue MYB8 in a herbivory-inducible fashion in wild tobacco, *N. attenuata* [18]. MYB8 (and MYBJS1) also likely contributes to lignin accumulation by targeting the control of the hydroxycinnamoyl-CoA:shikimate/quinate hydroxycinnamoyl transferase (HCT) gene in tobacco [19]–[21].

Because *PAP1*-overexpressing plants constitutively produce large amount of anthocyanins, we asked if the accumulation of other inducible flavonoids and phenylpropanoids was affected. PAP1 and MYBJS1 seem to share common gene targets in the pathways they regulate, and so these two transcription factors are likely to engage in complex interactions when *PAP1* is introduced into plants under a constitutive promoter and also, in response to herbivory, when other MYBs are additionally activated upon. It is likely that when *PAP1*-overexpressing plants are damaged by herbivores, the dynamic activation of the metabolic flow via other MYB members competes with activated coumaric acid units used in the constitutive synthesis of anthocyanins by PAP1-promoted enzymes. Both transcriptional and post-transcriptional regulations of PAP1 have been shown to control anthocyanin levels in ubiquitous light/dark rhythms [22]. Moreover, when anthocyanin production is regulated by independent PAP-transcription and environmental stress, anthocyanin levels are reduced [23]. Given that the stress of herbivory could affect PAP1 function in *PAP1*-overexpressing plants, we asked how efficiently transgenes such as PAP1 could be expected to perform under natural, uncontrolled environmental conditions.

In the current study, we found that the defensive properties of *PAP1*-overexpressing plants increased upon herbivore attack, and addressed the issue of metabolic fluxes and transcriptional regulation in the flavonoid/phenylpropanoid pathways that are co-regulated by powerful PAP1-activators and herbivory. Since jasmonic acid (JA) and its derivatives (referred as jasmonate [JAs]) coordinate the expression of regulatory and structural genes in the flavonoid/phenylpropanoid pathways under stress, we also assessed the role of JAs (and other phytohormones) in the herbivory- and PAP1-mediated regulation of phenylpropanoid metabolism. Finally, we discuss the implications of enhancing the production of specialized metabolites in plants under natural environmental conditions.

## Materials and Methods

### Production of transgenic tobacco plants

The full-length coding region of *Arabidopsis PAP1* (At1g56650) cDNA was inserted into the binary vector pSMABR35SsGFP by replacing the GFP reporter gene of the vector [24]. The resulting plasmid, pSMABR-35SPAP1, was transformed into *Agrobacterium tumefaciens* strain EHA101 by electroporation. *PAP1-*overexpressing tobacco (*N. tabacum* cv. SR1) was produced by *Agrobacterium*-mediated transformation as described previously [24]. After rooting and acclimatization, the regenerated plants were grown in a controlled greenhouse to set seeds. Nine lines of transgenic T_1_ seeds were tested for germination on Murashige and Skoog medium supplemented with 5 mg l^−1^ bialaphos at 25°C under a 16-h photoperiod at a light intensity of ca. 30 µE m^−2^ s^−1^. T_2_ seeds harvested from each T_1_ individual plant that showed ca. 3∶1 segregation ratio were tested for bialaphos-resistance again. Finally, T_2_ and T_3_ homozygous plant lines were used for further analyses. A homozygous tobacco T_2_ line transformed with the binary plasmid pSMABR-35SpGUS (in which the reporter *GFP* gene of pSMABR35SsGFP was replaced by β-glucuronidase [GUS] gene), expressing bialaphos resistance [bar] and GUS genes, was used as a control.

Three representative *PAP1*-overexpressing plants (*PAP1* lines), constitutively expressing *Arabidopsis PAP1* under the control of the cauliflower mosaic virus (CaMV) 35S promoter, were used. The *PAP1* lines exhibited substantial expression of *PAP1* based on their increased pigmentation throughout their development, in comparison to wild-type (WT) and transgenic plants expressing *uid*A (coding for the GUS reporter gene and serving as a control) (Figures S1 and S2). Except for having increased pigmentation, *PAP1* lines developed and matured in a similar manner to their control plants, as shown in previous studies [5], [6] (Figure S2).

### Plants and caterpillars

Tobacco plants were grown in plastic pots in a growth chamber at 25°C (16 h photoperiod at a light intensity of 80 µE m^−2^ s^−1^) for about 5 weeks. Eggs of *Spodoptera litura* (Fabricius) (Lepidoptera: Noctuidae) were obtained from Sumika Technoservice Co. Ltd. (Takarazuka, Japan), and the hatched larvae were reared on artificial diet (Insecta LF, Nihon Nosan Kogyo Ltd., Tokyo, Japan) in the laboratory at 25°C.

### Chemical and herbivore treatments and leaf sample preparation

For chemical treatment, methyl jasmonate (MeJA, 0.1 mM, Wako Pure Chemical Industrials, Ltd., Osaka, Japan) in 2 mL of 0.1% ethanol solution was sprayed onto intact plants in plastic pots. Ethanol solution (0.1%) was used as the control. For herbivore treatment, four third-instar larvae were incubated with a whole tobacco plant in a pot (Figure S3). All herbivory bioassays were carried out in a climate-controlled chamber at 25°C (16 h photoperiod). For the determination of phytochemical concentrations and RNA extractions, leaf tissue samples were harvested into the liquid nitrogen, flash-frozen, and kept at −80°C prior to extraction.

### Performance of *S. litura* larvae

Larvae were reared on artificial diet until the assay started, as described above. Second-instar *S. litura* larvae were weighed prior to release onto a potted plant (ranging from 0.7 to 1.2 mg) and released one at a time. Each larva was allowed to move on the whole plant in a climate-controlled room at 25°C under a 16-h photoperiod. Twenty-six to thirty-two larvae were analyzed for each line.

### Analysis of phenolic compounds

Phenylpropanoids and flavonoids in tobacco leaves were determined using high-performance liquid chromatography (HPLC) with a slight modification of the method described by Galis et al. (2013) and Matsuba et al. (2010) [25], [26]. Replicated analyses were conducted with five independent leaf samples.

For anthocyanin (cyanidin-3-rutinoside) determination, the ground leaf samples (300 mg each) were further homogenized in the liquid nitrogen and extracted with 3 ml of 80% methanol containing 1% trifluoroacetic acid (TFA) at 4°C overnight. After passing through a 0.22 µm syringe filter (Millipore, Billerica, MA, USA), filtrates (10 µl) were subjected to an HPLC (L-6320 Intelligent pump, L-4200 UV-VIS detector, AS-4010 Intelligent Autosampler, L-5025 column oven, Hitachi, Tokyo, Japan) equipped with a COSMOSIL 5C_18_-MS-II packed column (4.6 mm×50 mm; Nacalai Tesque, Kyoto, Japan). The separation was performed by the linear gradient elution of 10–70% (v/v) methanol in 1.5% aqueous phosphoric acid for 5 min at a flow rate of 1.5 ml min^−1^ at 35°C. The elution profiles were monitored at 528 nm and recorded using the Chromato-Pro (Runtime Instruments, Kanagawa, Japan). The authentic compound used for quantification was cyanidin-3-rutinoside (Funakoshi Co. Ltd., Tokyo, Japan).

To determine phenylpropanoids (chlorogenic acid) and flavonoids (kaempferol 3-*O*-rutinoside and rutin), the ground leaf samples (100 mg each) were briefly homogenized in the liquid nitrogen at room temperature in 0.75 ml of 84 mM acetate buffer (adjusted to pH 4.8 with ammonia) in 40% methanol (v/v) using a FastPrep FP120 instrument (Thermo Savant, Thermo Fisher Scientific, Pittsburgh, PA, USA) in the presence of 2.3-mm diameter zirconia beads. Samples were centrifuged for 20 min at 16,100 g, 4°C, and supernatants were collected. Pellets were re-extracted in 0.75 ml buffer containing 80% (v/v) methanol by shaking at RT for 15 min and centrifuged as before; supernatants were pooled with the first extracts. Aliquots (20 µl) of each sample were analyzed on a Shimadzu Prominence HPLC system equipped with a COSMOSIL 5C_18_-MS-II packed column (4.6 mm×50 mm; Nacalai Tesque, Kyoto, Japan) in a gradient setup: solvent A (0.1% formic acid, 0.1% ammonia in water, pH 3.6) and solvent B (methanol); time (min)/B (%) gradient conditions: 0/2, 10/80, 15/80, 16/95, 21/95, 24/2, followed by a 6-min equilibration step. Flow rate was set to 1 ml/min and the column was maintained at 35°C. Chromatographic peaks of chlorogenic acid isomers and rutin were detected by an SPD-M20A diode array detector and compared with external calibration curves of authentic chlorogenic acid (Sigma-Aldrich) and rutin (Ishizu Pharmaceuticals Co. Ltd., Osaka, Japan) standards, respectively. Kaempferol 3-*O*-rutinoside content was estimated based on rutin external calibration.

### Identification of phenolic acid and flavonoid compounds

Leaf extracts of *PAP1* lines originally prepared for HPLC analyses were used to identify the major UV-absorbing peaks in chromatograms. A 20-µl aliquot of leaf sample was first separated on HPLC as above, and peak fractions of major UV-absorbing compounds were collected using an automated fraction collector (FRC-10A, Shimadzu, Kyoto, Japan). Fractions from 10 runs were pooled, evaporated, re-dissolved in 70% methanol in water (v/v) and spun shortly before analysis. A 10-µl aliquot of each fraction was subjected to measurement on a triple quadrupole liquid chromatography-tandem mass spectrometry (MS) system (LC-MS/MS 6410, Agilent Technologies, Santa Clara, CA, USA) equipped with a Zorbax SB-C18 column (2.1 mm id×50 mm, (1.8 µm), Agilent Technologies), maintained at 40°C and coupled to an MS detector operating in positive scan mode (m/z 100–1000; fragmentor at 135 V). Samples were passed through a UV-PDA detector before entering the MS electrospray ion source for reference and later identification of the peaks. Solvent A (0.1% formic acid in water) and solvent B (0.1% formic acid in acetonitrile) were used in time (min)/B (%) gradient mode: 0/5, 0.5/5, 2/40, 6/40, 10/95, 15/95, 16/5, 20/5 at a constant flow rate of 0.4 ml/min. The mass spectra corresponding to UV-absorbing peaks in the chromatograms were analyzed and used to identify each compound. Only the rutin peak could be assigned against an authentic rutin standard (Ishizu Pharmaceutical Co., Ltd., Osaka, Japan), while the remaining peaks were putatively assigned based on their m/z, fragmentation properties and characteristic UV absorbance spectra.

### Phytohormone analysis

Leaf hormone concentrations were determined according to the method described by Fukumoto et al. (2013) [27]. Approximately 100 mg of the ground leaf samples was further homogenized in liquid nitrogen and extracted in 1 ml of ethylacetate spiked with internal standards (IS): 25 ng d3-JA; 5 ng d3-JA-Ile; 10 ng d6-ABA and 10 ng d4-SA. After brief homogenization in a FastPrep instrument as above, samples were centrifuged at 4°C, 20 min at 16,100 *g*. Supernatants were stored and pellets re-extracted in 0.5 ml of ethylacetate without IS. After centrifugation, pooled supernatants were evaporated under reduced pressure in a rotary vacuum concentrator and re-suspended in 250 µl of 70% methanol/water (v/v). Ten-microliter aliquots were analyzed on a triple quadrupole LC-MS/MS 6410 equipped with a Zorbax SB-C18 chromatographic column [2.1 mm id×50 mm, (1.8 µm), Agilent Technologies] protected by a narrow bore guard column Zorbax SB-C8 [2.1 mm id×12.5 mm, (5 µm)]. Samples were chromatographically resolved using solvents A [0.1% formic acid in water (v/v)] and B [0.1% formic acid in acetonitrile (v/v)] used in the following gradient: time (min)/B (%) 0/15, 4.5/98, 12/98, 12.1/15, 18/15. Flow rate was set to 0.4 ml/min and column temperature maintained at 40°C. Mass transitions for each compound [hormone/Q1 precursor ion (m/z)/Q3 product ion (m/z)] were detected in the ESI-negative mode of the mass spectrometer: JA/209/59; JA-Ile/322/130; ABA/263/153; SA/137/93; d3-JA/212/59; d3-JA-Ile/325/130; d6-ABA/269/159; d4-SA/141/97. Hormone amounts were calculated from the ratio of endogenous hormone peak and known amount of IS spike, and adjusted to the exact fresh mass (FM) of the sample used for extraction. Replicated analyses were conducted with five independent leaf samples.

### cDNA synthesis and quantitative PCR

Approximately 100 mg of the ground leaf samples was homogenized in liquid nitrogen, and total RNA was isolated from leaf tissues using NucleoSpin RNA Plant (Machery-Nargel, Düren, Germany) following the manufacturer's protocol. First-strand cDNA was synthesized using a ReverTra Ace qPCR RT Master mix (Toyobo, Osaka, Japan) and 0.5 µg of total RNA at 37°C for 15 min. Real-time PCR was performed on a Light Cycler Nano system (Roche Applied Science, Indianapolis, IN, USA) using FastStart Essential DNA Green Master (Roche Applied Science) and gene-specific primers (Table S1). The following protocol was used: initial polymerase activation: 60 s at 95°C; 40 cycles of 15 s at 95°C and 45 s at 60°C; and then melting curve analysis preset by the instrument. Relative transcript abundances were determined after normalization of raw signals with housekeeping transcript abundances of *N. tabacum ELONGATION FACTOR 1α* (*NtEF1α*; GenBank D63396). Replicated analyses were conducted with four to five independent leaf samples.

### Statistical analysis

All experiments were repeated at least four times, and statistical analyses were performed using a Tukey's HSD test after a one-way ANOVA. A *P* value<0.05 was considered statistically significant.

## Results

### Transgenic tobacco plants constitutively expressing *PAP1* genes are better able to defend themselves against herbivores than are non-transgenic tobacco plants

**Figure 1:**
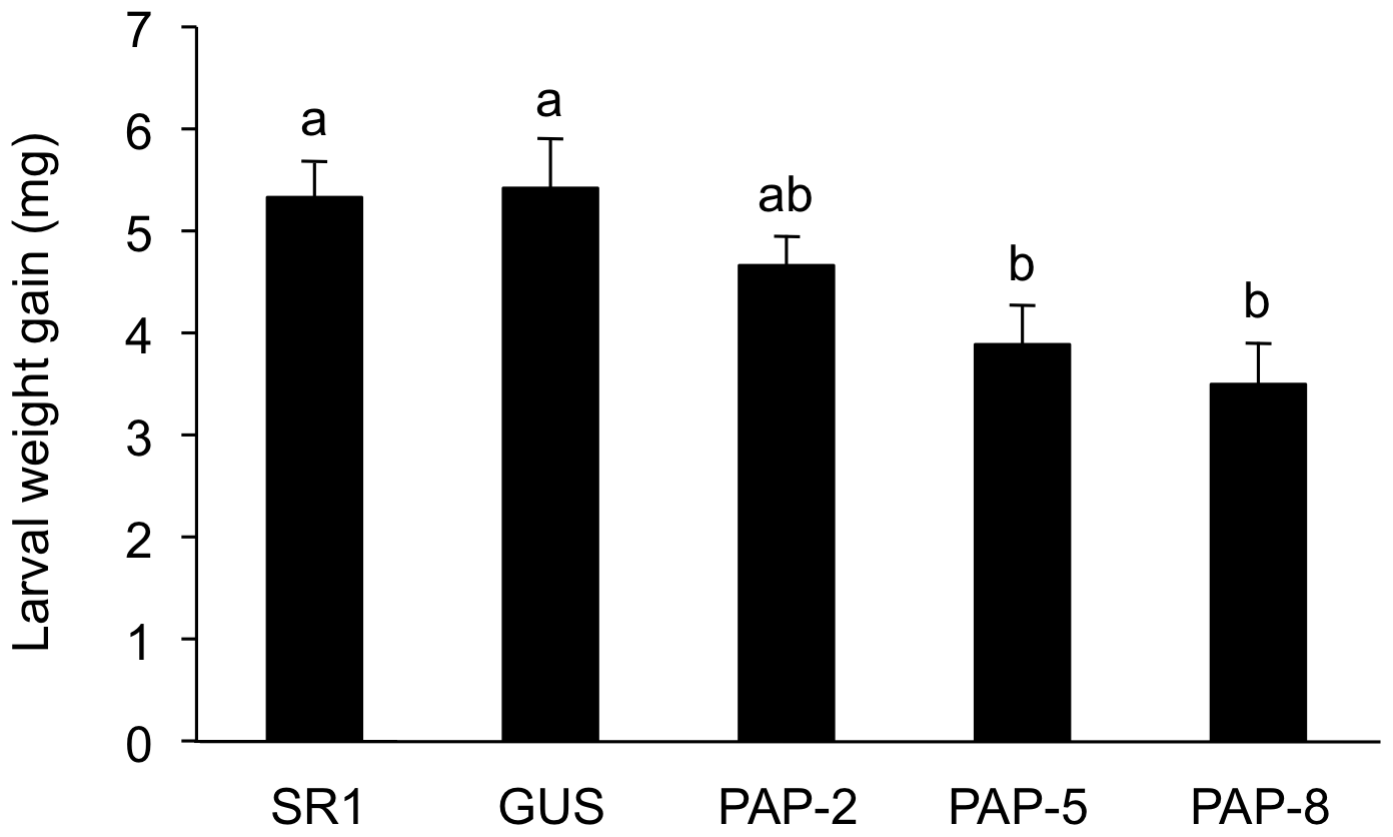
The net body weight that *Spodoptera litura* larvae gained during 4 days on wild-type (WT), GUS control and *PAP1* lines. Data are shown as the mean+standard errors (*n* = 26–32). Means followed by different small letters are significantly different (*P*<0.05).

We evaluated the resistance of *PAP1* lines, constitutively expressing *Arabidopsis PAP1*, to the arthropod herbivore *S. litura*. Larvae were applied onto the leaves of potted WT, GUS and *PAP1* lines for 2 days (Figure 1). Larvae on the PAP-5 and PAP-8 lines had markedly lower weight gain compared to those on WT or GUS lines (*P*<0.05). Larvae on another *PAP1* line (PAP-2) exhibited slightly, but not significantly, less weight gain compared to those on WT or GUS lines (*P*>0.05).

### Biosynthesis of flavonoids/phenylpropanoids in *PAP1* lines

**Figure 2:**
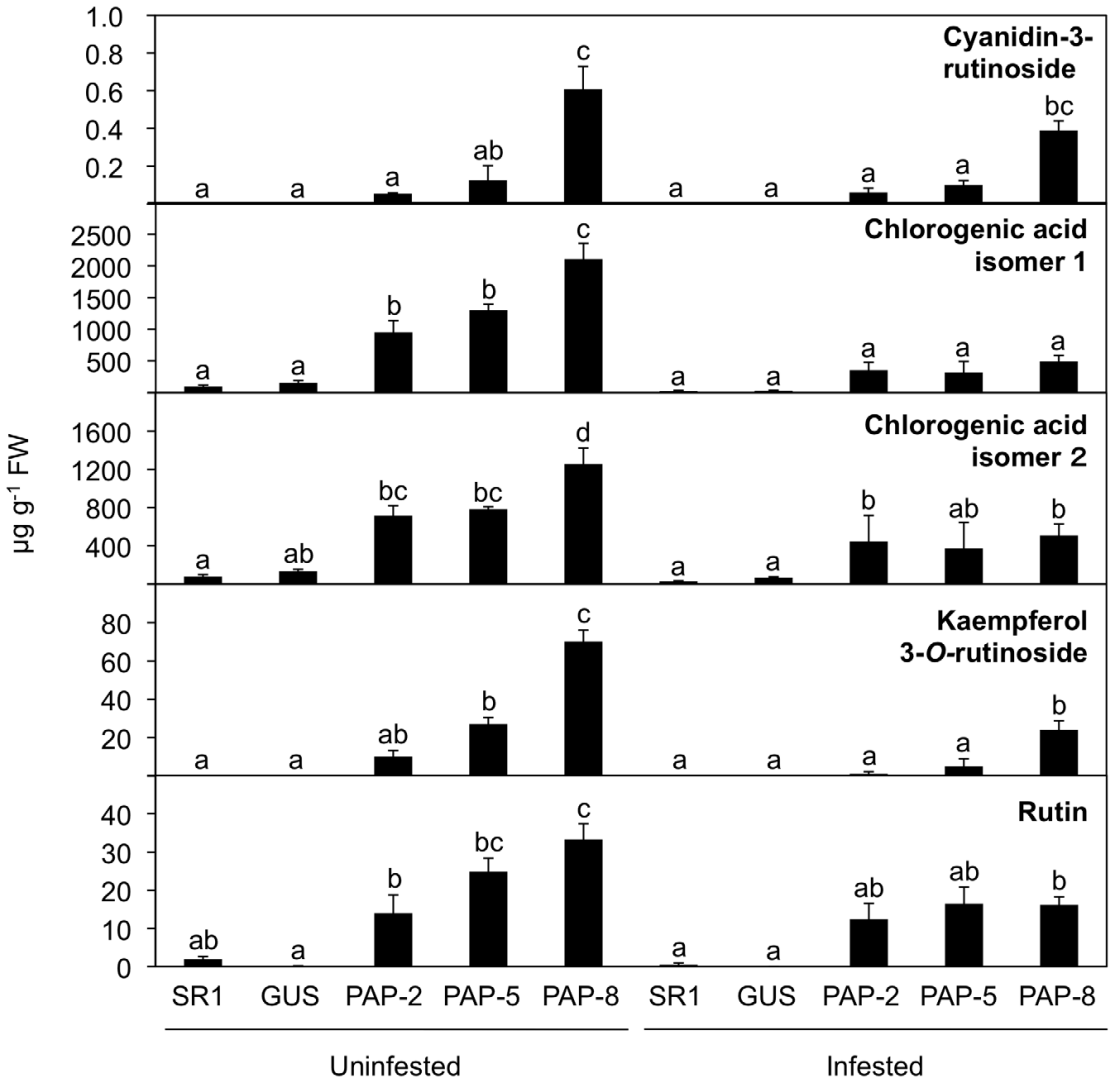
Endogenous accumulation of flavonoids/phenylpropanoids. Values for the major anthocyanin compound (cyanidin-3-*O*-rutinoside), other flavonoid (kaempferol 3-*O*-rutinoside and rutin) and phenylpropanoid (chlorogenic acid) compounds were determined in leaves of wild-type (WT), GUS control and *PAP1* lines not infested or infested with *Spodoptera litura* for 2 days. Data are shown as the mean+standard error (*n* = 5). Means followed by different small letters are significantly different (*P*<0.05).

Since PAP1 has been shown to activate the phenylpropanoid/flavonoid pathway [28], we measured endogenous levels of their metabolites in *PAP1* lines. According to HPLC analysis supported by identification of the most abundant compounds by LC-MS/MS, there was increased accumulation of an anthocyanin compound (cyanidin-3-*O*-rutinoside) as well as of two other flavonoid compounds (kaempferol 3-*O-*rutinoside and rutin) and of phenylpropanoid compounds (chlorogenic acid isomers 1 and 2) in leaves of *PAP1* lines, relative to WT and GUS lines (Figure 2). PAP-8 leaves accumulated all of the above five compounds at the highest levels, unsurprisingly as this line was best able to defend itself against *S. litura* larvae (see Figure 1). Interestingly, *S. litura* infestation substantially diminished the increase of the concentration of these compounds in *PAP1* lines, whereas no significant change (either increase or decreased) of those compounds was observed in both WT and GUS lines (Figure 2).

**Figure 3:**
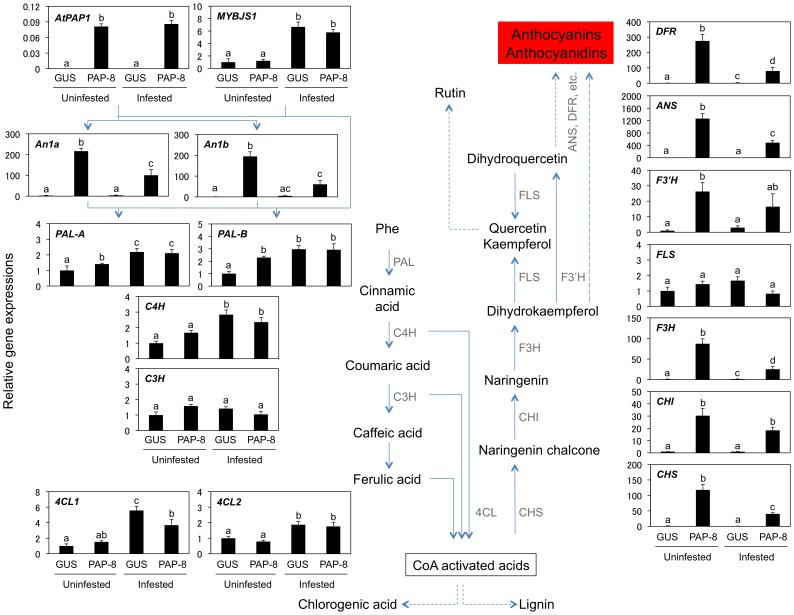
Expression of genes for transcription factors PAP1 and MYBJS1, An1a, An1b and structural genes involved in the flavonoid/phenylpropanoid pathway. Relative transcript levels of genes were determined in leaves of GUS control and *PAP1* lines (PAP-8) not infested or infested with *Spodoptera litura* for 2 days. Transcript levels of genes were normalized by those of *NtEF1α*. Data are shown as the mean+standard errors (*n* = 4–5). Means followed by different small letters are significantly different (*P*<0.05). Expression of genes in GUS and PAP-8 leaves after 4 days of damage are presented in Figure S4. ANS, anthocyanidin synthase; C3H, cinnamate 3-hydroxylase; C4H, cinnamate 4-hydroxylase; CHI, chalcone isomerase; CHS, chalcone synthase; 4CL, 4-coumarate coenzyme A ligase; DFR, dihydroflavonol 4-reductase; F3H, flavanone 3-hydroxylase; F3′H, flavonoid 3′-hydroxylase; FLS, flavonol synthase; PAL, phenylalanine ammonia-lyase.

We therefore assessed the transcriptional pattern of flavonoid/phenylpropanoid pathway genes in leaves of *PAP1* lines not infested or infested with *S. litura* for 2 days (Figure 3). In comparison to GUS lines, PAP-8 showed significantly increased transcript levels of eight genes (*PALs*, *CHS*, *CHI*, *F3H*, *F3′H*, *ANS* and *DFR*) involved in phenylpropanoid and flavonoid biosynthesis. The increase of the transcript level was, however, diminished by 34, 60, 29, 39 and 29% of the initial increase in five cases, *CHS*, *CHI*, *F3H*, *ANS* and *DFR*, respectively, in *PAP1* lines in response to herbivory. *PAP1* was expressed similarly between uninfested and infested PAP-8 leaves. Moreover, we also determined the transcript levels of four genes responsible for producing endogenous transcription factors: MYBJS1 (another MYB transcription factor sharing transcriptional targets with PAP1 in the phenylpropanoid pathway [18]), An2 (PAP1 homologue MYB transcription factor [29]), and An1a and An1b (NtAn2-interacting, basic helix-loop-helix (bHLH) transcription factors [30]). An1a, An1b and An2 are known to be responsible for anthocyanin pigmentation in tobacco flowers [29], [30]. The transcript of *MYBJS1* was induced similarly between GUS and PAP-8 leaves indicates that the *MYB* gene was not under the direct control of PAP1. *An2* was not expressed in either GUS or PAP-8 leaves, confirming what was previously known, namely, that this transcription factor is abundantly expressed in flowers but not in leaves [29]. On the other hand, although PAP-8 increased transcript levels of *An1a* and *An1b* transcript levels of GUS lines, the increase of the transcript levels was diminished by herbivory; this result was also observed in the structural genes mentioned above (see Figures 3 and S4 for 2 days and 4 days of exposure, respectively).

### Upstream and downstream JA signaling for *PAP1*-activated genes

**Figure 4:**
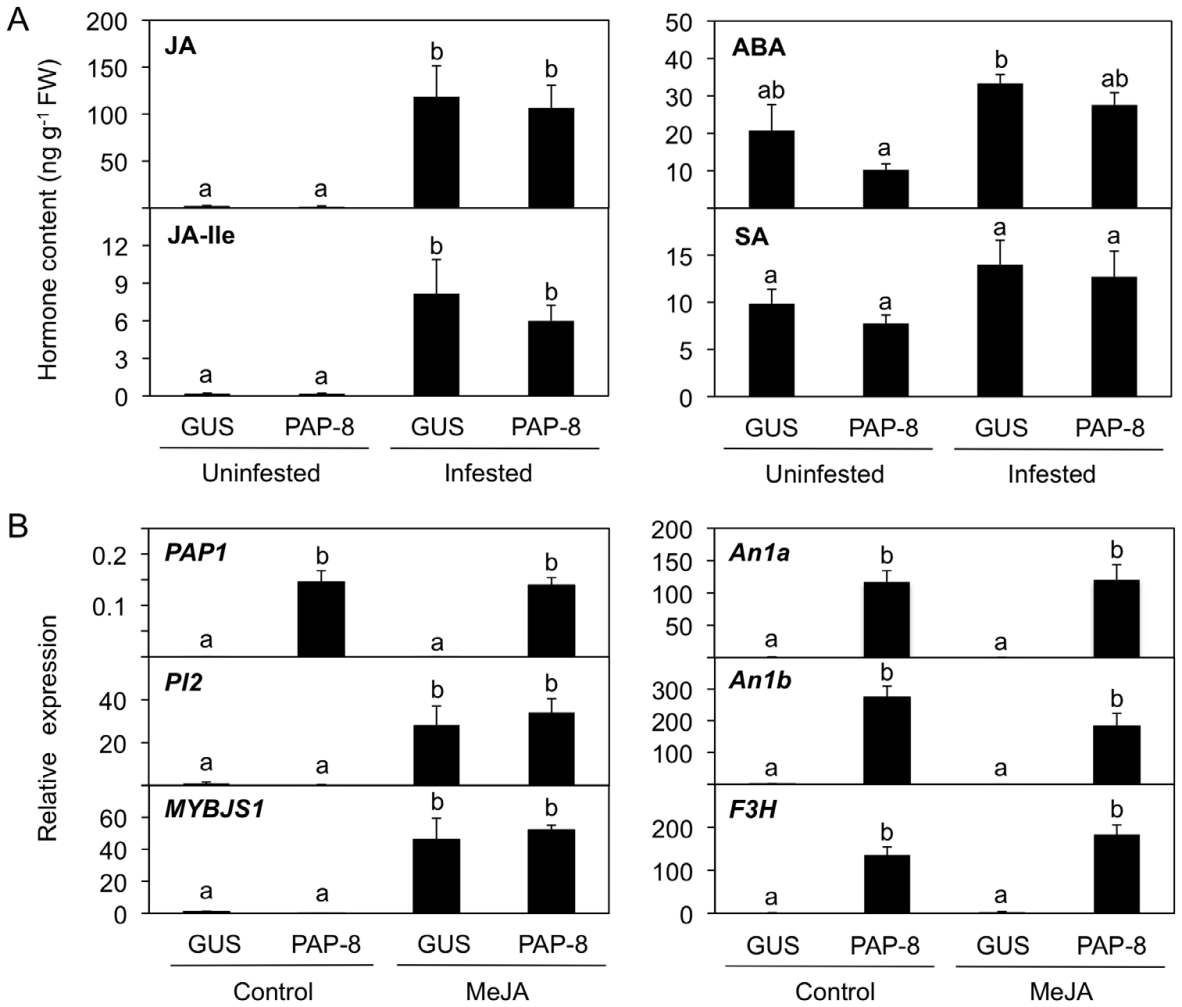
Upstream and downstream transcription regulation of PAP1-activated genes. A, Endogenous phytohormone levels were determined in leaves of GUS control and *PAP1* lines (PAP-8) not infested or infested with *Spodoptera litura* for 2 days. ABA, abscisic acid; JA, jasmonic acid; JA-Ile, jasmonoyl-L-isoleucine; SA, salicylic acid. B, The effect of treatment with methyl jasmonate (MeJA) on gene expression was determined in leaves of GUS and PAP-8 lines treated with 0.1 mM MeJA (in 0.1% ethanol solution) for 24 h. Plants treated with 0.1% ethanol solution for 24 h served as controls. Transcript levels of genes were normalized by comparing them to those of *NtEF1α*. Data are shown as the mean+standard error (*n* = 4–5). Means followed by different small letters are significantly different (*P*<0.05). F3H, flavanone 3-hydroxylase; PI2, proteinase inhibitor 2.

Since JA signaling along with other phytohormone signaling plays a central role in defense responses against chewing herbivores [31], it might be expected that induced JA production and signaling would be relevant to the low concentration of flavonoid/phenylpropanoid pathway products in *PAP1* lines attacked by *S. litura*. To assess whether *PAP1* overexpression affects JA biosynthesis and signaling, we analyzed endogenous levels of jasmonoyl-L-isoleucine (JA-Ile, an active form of JAs [32]) and of two other phytohormones: abscisic acid (ABA, involved in protective wound-healing processes [33]) and salicylic acid (SA, involved in pathogenesis and herbivore responses [34]–[36]). As shown in Figure 4A, JA and JA-Ile increased and accumulated at similar rates in the infested PAP-8 leaves compared to in the leaves of infested GUS lines. Those data indicate that PAP1 is not an upstream transcription regulator for the biosynthesis of JA. Moreover, neither ABA nor SA was increased in leaves of GUS or PAP-8 lines upon infestation, indicating that neither of those two hormones is directly linked by PAP1 actions.

Next, we investigated the gene expression levels in leaves of GUS and PAP-8 lines by applying an exogenous solution of MeJA (the commonly used active form of JAs [37]: 0.1 mM). Even though the PAP1-regulated transcript levels of *An1a*, *An1b* and *F3H* are negatively affected by herbivory (as shown in Figure 3), MeJA did not reduce their transcript levels to the same extent that it reduced transcript levels of *PAP1* (Figure 4B). The MeJA concentration used for our treatment was undoubtedly sufficient to induce defense responses in leaf tissues, as the transcript levels of a typical MeJA-inducible protease inhibitor (PIs [38]) and *MYBJS1* genes were substantially up-regulated in the same treated samples. We therefore conclude that the down-regulation of PAP1-activated genes after herbivory is closely linked not to the JA signaling and biosynthesis cascades but, rather, to other herbivory-associated signals discussed below.

### Reversible and transient regulatory patterns of *F3H* and *PI2* during herbivory and post-herbivory periods

**Figure 5:**
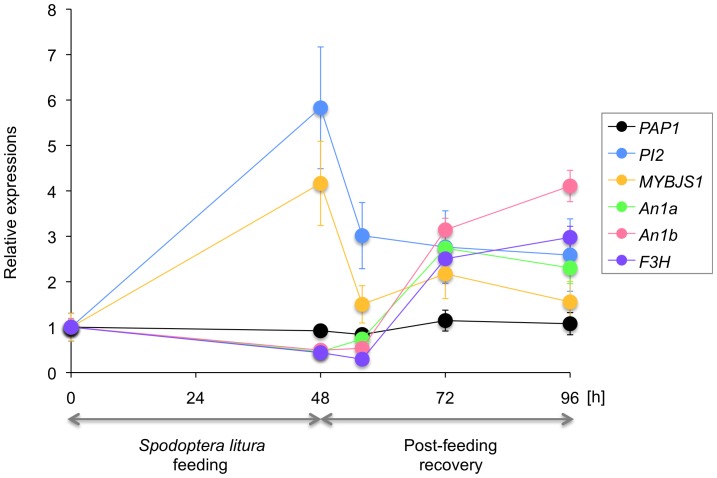
Temporal patterns of *F3H* and *PI2* gene regulation in leaves of *PAP1* lines (PAP-8) plants during exposure to *Spodoptera litura* for 48 h and for up to another 48 h after removal of the larvae (recovery period). Transcript levels of genes were normalized by comparing them to those of *NtEF1α*. Data are shown as the means ± standard errors (*n* = 4–5).

To clarify the temporal regulation of PAP1-regulated genes, we analyzed the temporal patterns of the expression levels of *F3H* (PAP1-regulated) and *PI2* (JA-inducible) not only during 48 h after the first exposure to *S. litura* but also for up to 48 h after the larvae were removed (post-herbivory recovery; Figure 5). Consistent with the data shown in Figure 3, the regulation of the expression levels of *An1a*, *An1b* and *F3H* by PAP1 was suppressed 48 h after the first exposure to *S. litura*. After the larvae were removed, the expression levels remained low during the first 8 h but then increased 24 h and 48 h after the damage had ended. In contrast, *PI2* and *PAP1* displayed different expression profiles: the expression level of *PI2* reached a maximum at 48 h after first exposure to *S. litura* but dropped to the basal level 8 h, 24 h and 48 h after the damage had ended, consistent with the defense function of the PI2 protein; the expression level of *PAP1* was unchanged through the experimental period. Collectively, these results suggest that *F3H* might be regulated positively by an additional, JA-independent regulator(s) during the post-herbivory period.

## Discussion

To date, light stimuli and abiotic stress have been reported to modulate anthocyanin concentrations in several plant taxa. For instance, in grape berries, and flowers of rose and *Brunsfelsia calycina*, anthocyanin accumulation appears to be suppressed in response to high temperature [39]–[41]. Most impressively, Rowan et al. (2009) reported that abiotic stress treatments of high temperature and low light cause reductions in the concentrations of anthocyanins and down-regulation of the genes involved in anthocyanin biosynthesis in leaves of *PAP1*-overexpressing *Arabidopsis* plants [23]. The inhibition of transcription occurs independently of *PAP1*, and the loss of anthocyanins is not the result of an obvious catabolic enzyme regulated at the transcription level. The authors addressed whether the decreased flux of anthocyanins into the vacuole occurred as a consequence of a decline in the expression of biosynthetic genes by reducing the expression of three genes (*TT8*, *TTG1* and *EGL3*) of the PAP1 transcriptional complex and enhancing the expression of the potential transcriptional repressors *AtMYB3*, *AtMYB6* and *AtMYBL2*. Those results especially reflect to our finding that transcript levels of both biosynthetic genes and endogenous transcription factor (positive regulator) genes were decreased in infested PAP1 plants compared to those in the uninfested PAP1 plants (Figure 3). *An1a* and *An1b* function by interacting with *An2* (PAP1 homologue) to regulate anthocyanin biosynthesis [30], thus implying that those two factors can also interact with *Arabidopsis* PAP1. In fact, *An1a* and *An1b* transcripts were enhanced in the overexpression of *PAP1*, suggesting that those two bHLH proteins are able to interact with foreign PAP1 genes and are involved in the feedback regulation by the MYB/bHLH complexes as reported in *Arabidopsis* [42]. The diminished expression of those two genes suppress the expression of structural genes – genes that are also suppressed by herbivory – and regulates the flux of flavonoid/phenylpropanoid (Figures 2 and 3). Presumably, in return, plants gain energy and the resources required for induced defense responses (so-called homeostasis and feedback regulation), which might eventually make plants better able to withstand to generalist herbivores.

**Figure 6:**
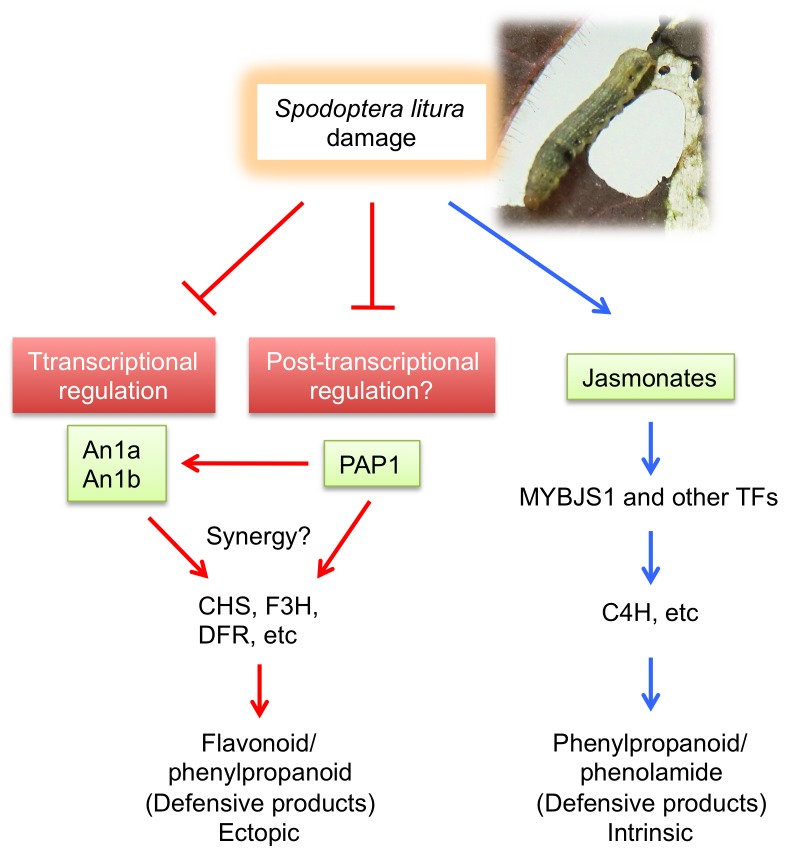
Schematic presentation of effect of *Spodoptera litura* feeding on the PAP1-regulated phenylpropanoid biosynthetic genes in a manner independent of jasmonate signaling. Arrows and bars indicate positive and negative interactions, respectively. TFs, transcription factors.

We present here two important findings, namely, that *PAP1* lines that accumulated great amounts of flavonoid/phenylpropanoid pathway products defend against the generalist herbivore *S. litura*, and that the *PAP1*-stimulated accumulations of those compounds are attenuated by herbivory (Figure 6). The first finding is in line with a previous report that transgenic tobacco plants expressing *Arabidopsis* transcription factor *AtMYB12* exhibit more resistance to *S. litura* and *Helicoverpa armigera* than do non-transgenic plants, most probably due to these plants' enhanced accumulation of rutin [13], a chemical that is toxic for those herbivores [43]. As rutin is one of predominant products in *PAP1* lines (Figure 2), this flavonoid compound is predicted to be a major anti-herbivore agent in our case as well.

The suppression of flavonoid/phenylpropanoid pathway products by herbivory is likely to reflect the phenomena described above, such as the response of *Arabidopsis* plants to abiotic stress, namely, the overexpression of *PAP1* [23]. In addition to the proposed reason for suppression (see above), we should consider an alternative (or additional) explanation, such as that PAP1 protein could be post-transcriptionally degraded under herbivory even though normal transcript levels of *PAP1* in PAP-8 leaves are maintained during herbivory (Figure 3). A similar mechanism is consistent with the repression of anthocyanin in the dark; in this case, the COP1/SPA ubiquitin ligase plays a critical role in the degradation of PAP1 proteins [22]. Although we conducted immunoblot analysis using polyclonal antibodies against partial PAP1 peptide residues ([H]CKIKMKKRDITP[OH]) (produced by Sigma-Aldrich, Ishikari, Japan), our efforts unfortunately failed to detect sufficient PAP1 protein signals extracted from uninfested and infested PAP-8 leaves. Therefore, it remains to be clarified whether PAP1 degradation in response to herbivory is subject to similar mechanisms for light/dark responses observed in *Arabidopsis*.

Alternatively, the possibility that the general decrease observed in the concentration of phenolic compounds after two days of herbivory could be a result of an altered metabolic precursor allocation (competition) cannot be excluded. For example, in transgenic *N. attenuata* plants, the transient silencing of the expression of a hydroxycinnamoyl-CoA:shikimate/quinate hydroxycinnamoyl transferase (HCT) gene involved in lignin biosynthesis dramatically increased herbivory-induced phenolamides produced in the parallel metabolic branch [21]. It was proposed that a strong metabolic tension in the plant, exacerbated during herbivory, exists over the allocation of coumaroyl-CoA units among lignin and other phenolic compounds. This tension could be extended to anthocyanins and flavonoids, as shown in our experiments (see Figure 2), although the branches that are simultaneously enhanced have not yet been identified. In addition, the enhanced catabolism of some phenolics during herbivory stress could be responsible. Unfortunately, very little is known about the anthocyanins, kaempferol-3-*O*-rutinoside and rutin catabolism in plants, and the hypothesis that there is competition among metabolic precursors remains to be tested.

Generally, since JA-mediated defense signaling is predominantly activated when plants are attacked by chewing herbivores [31], it might be expected that induced JA production and signaling would be relevant to the defense responses to *S. litura* attack. Even though *PI2* expression was highly induced during herbivory and exogenous MeJA application (see Figures 3 and 4), it was likely that the *PAP1*-activated genes were suppressed independently of the induction of JA and JA-Ile (an active form of JA) (Figure 4A). Moreover, since MeJA application also failed to suppress PAP1-activated genes in PAP-8 plants (Figure 4B), JA signaling is not likely a part of the down-regulation pathway. Also, in our case SA and ABA signaling pathways seem to be irrelevant in our case (see Figure 4A). Those facts may reflect a recent report that a mitogen-activated protein kinase (MPK4) suppresses a JA-independent defense pathway in *N. attenuata* in response to *Manduca sexta* feeding [44].

Moreover, the reverse mechanism was also likely used, given that PAP1-regulated *F3H* expression was dramatically increased in PAP-8 leaves during the post-herbivory period, when JA signaling was already switched off (Figure 5). This up-regulation might be caused by the fine-tuning of the PAP1 protein level after the cessation of herbivory, resulting in increased PAP1 levels beyond the basal level in the undamaged condition. Alternatively, additional signal(s) might be facilitated only when JA signaling is turned off, although these speculations require further investigation.

Horticultural ecosystems are very complicated and flexible. Hence, prior to setting genetically modified plants in the field, a wide range of systematic studies should be conducted to understand realistic horticultural ecosystems, where a myriad of plants, animals, and microorganisms interact and coevolve. However, it still remains to be elucidated how other multiple and simultaneous stresses can potentially influence flavonoid/phenylpropanoid catabolism in real agricultural settings.

## Supporting Information

Figure S1 **Relative mRNA levels of** ***PAP1*** **in leaves of wild-type (WT), GUS and** ***PAP1*** **lines.** Transcript levels of genes were normalized by those of *NtEF1α*. Data are shown as the mean+standard errors (*n* = 5). ND, not detected.
(TIF)

Figure S2 **Morphology of** ***PAP1*** **lines.** A, 4-week-old plants; B, 8-week-old plants; C, flowers.
(TIF)

Figure S3 **GUS and PAP-8 plants infested with** ***Spodoptera litura*** **for 2 days (A) and 4 days (B).**
(TIF)

Figure S4 **Expression of genes in GUS and PAP-8 leaves infested with** ***Spodoptera litura*** **for 4 days.** Relative transcript levels of genes were determined in leaves of GUS control and *PAP1* lines (PAP-8) not infested or infested with *S. litura* for 4 days. Transcript levels of genes were normalized by comparing them to those of *NtEF1α*. Data are shown as the mean+standard errors (*n* = 4–5). Means followed by different small letters are significantly different (*P*<0.05).
(TIF)

Table S1 **RT-PCR primers used for this study.**
(XLSX)

## References

[1] Dixon RA, Liu C, Jun JH (2013) Metabolic engineering of anthocyanins and condensed tannins in plants. Curr Opin Biotechnol 24: 329–335. 22901316 10.1016/j.copbio.2012.07.004

[2] Glenn WS, Runguphan W, O'Connor SE (2013) Recent progress in the metabolic engineering of alkaloids in plant systems. Curr Opin Biotechnol 24: 354–365. 22954587 10.1016/j.copbio.2012.08.003 PMC3552043

[3] Wang Y, Chen S, Yu O (2011) Metabolic engineering of flavonoids in plants and microorganisms. Appl Microbiol Biotechnol 91: 949–956. 21732240 10.1007/s00253-011-3449-2

[4] Borevitz JO, Xia Y, Blount J, Dixon RA, Lamb C (2000) Activation tagging identifies a conserved MYB regulator of phenylpropanoid biosynthesis. Plant Cell 12: 2383–2394. 11148285 10.1105/tpc.12.12.2383 PMC102225

[5] Xie DY, Sharma SB, Wright E, Wang ZY, Dixon RA (2006) Metabolic engineering of proanthocyanidins through co-expression of anthocyanidin reductase and the PAP1 MYB transcription factor. Plant J 45: 895–907. 16507081 10.1111/j.1365-313X.2006.02655.x

[6] Ben Zvi MM, Shklarman E, Masci T, Kalev H, Debener T, et al (2012) *PAP1* transcription factor enhances production of phenylpropanoid and terpenoid scent compounds in rose flowers. New Phytol 194: 430–439. 22548501 10.1111/j.1469-8137.2012.04161.x

[7] Ben Zvi MM, Negre-Zakharov F, Masci T, Ovadis M, Shklarman E, et al (2008) Interlinking showy traits: co-engineering of scent and colour biosynthesis in flowers. Plant Biotechnol J 6: 403–415. 18346094 10.1111/j.1467-7652.2008.00329.x

[8] Zhang Y, Yan YP, Wang ZZ (2010) The *Arabidopsis* PAP1 transcription factor plays an important role in the enrichment of phenolic acids in *Salvia miltiorrhiza* . J Agric Food Chem 58: 12168–12175. 21058651 10.1021/jf103203e

[9] Mellway RD, Tran LT, Prouse MB, Campbell MM, Constabel CP (2009) The wound-, pathogen-, and ultraviolet B-responsive *MYB13*4 gene encodes an R2R3 MYB transcription factor that regulates proanthocyanidin synthesis in poplar. Plant Physiol 150: 924–941. 19395405 10.1104/pp.109.139071 PMC2689947

[10] Korkina LG (2007) Phenylpropanoids as naturally occurring antioxidants: from plant defense to human health. Cell Mol Biol 53: 15–25. 17519109

[11] Chalker-Scott L (1999) Environmental significance of anthocyanins in plant stress responses. Photochem Photobiol 70: 1–9.

[12] Kumar V, Nadda G, Kumar S, Yadav SK (2013) Transgenic tobacco overexpressing tea cDNA encoding dihydroflavonol 4-reductase and anthocyanidin reductase induces early flowering and provides biotic stress tolerance. PLoS ONE 8: e65535. 23823500 10.1371/journal.pone.0065535 PMC3688816

[13] Misra P, Pandey A, Tiwari M, Chandrashekar K, Sidhu OP, et al (2010) Modulation of transcriptome and metabolome of tobacco by Arabidopsis transcription factor, *AtMYB12*, leads to insect resistance. Plant Physiol 152: 2258–2268. 20190095 10.1104/pp.109.150979 PMC2850017

[14] Gális I, Simek P, Narisawa T, Sasaki M, Horiguchi T, et al (2006) A novel R2R3 MYB transcription factor NtMYBJS1 is a methyl jasmonate-dependent regulator of phenylpropanoid-conjugate biosynthesis in tobacco. Plant J 46: 573–592. 16640595 10.1111/j.1365-313X.2006.02719.x

[15] Kaur H, Heinzel N, Schöttner M, Baldwin IT, Gális I (2010) R2R3-NaMYB8 regulates the accumulation of phenylpropanoid-polyamine conjugates, which are essential for local and systemic defense against insect herbivores in *Nicotiana attenuata* . Plant Physiol 152: 1731–1747. 20089770 10.1104/pp.109.151738 PMC2832263

[16] Bassard JE, Ullmann P, Bernier F, Werck-Reichhart D (2010) Phenolamides: bridging polyamines to the phenolic metabolism. Phytochemistry 71: 1808–1824. 20800856 10.1016/j.phytochem.2010.08.003

[17] Gulati J, Baldwin IT, Gaquerel E (2014) The roots of plant defenses: Integrative multivariate analyses uncover dynamic behaviors of roots' gene and metabolic networks elicited by leaf herbivory. Plant J 77: 880–892. 24456376 10.1111/tpj.12439 PMC4190575

[18] Onkokesung N, Gaquerel E, Kotkar H, Kaur H, Baldwin IT, et al (2012) MYB8 controls inducible phenolamide levels by activating three novel hydroxycinnamoyl-coenzyme A:polyamine transferases in *Nicotiana attenuata* . Plant Physiol 158: 389–407. 22082505 10.1104/pp.111.187229 PMC3252090

[19] Hoffmann L, Besseau S, Geoffroy P, Ritzenthaler C, Meyer D, et al (2004) Silencing of hydroxycinnamoyl-coenzyme A shikimate/quinate hydroxycinnamoyltransferase affects phenylpropanoid biosynthesis. Plant Cell 16: 1446–1465. 15161961 10.1105/tpc.020297 PMC490038

[20] Hoffmann L, Maury S, Martz F, Geoffroy P, Legrand M (2003) Purification, cloning, and properties of an acyltransferase controlling shikimate and quinate ester intermediates in phenylpropanoid metabolism. J Biol Chem 278: 95–103. 12381722 10.1074/jbc.M209362200

[21] Gaquerel E, Kotkar H, Onkokesung N, Galis I, Baldwin IT (2013) Silencing an *N*-acyltransferase-like involved in lignin biosynthesis in *Nicotiana attenuata* dramatically alters herbivory-induced phenolamide metabolism. PLoS ONE 8: e62336. 23704878 10.1371/journal.pone.0062336 PMC3660383

[22] Maier A, Schrader A, Kokkelink L, Falke C, Welter B, et al (2013) Light and the E3 ubiquitin ligase COP1/SPA control the protein stability of the MYB transcription factors PAP1 and PAP2 involved in anthocyanin accumulation in Arabidopsis. Plant J 74: 638–651. 23425305 10.1111/tpj.12153

[23] Rowan DD, Cao M, Lin-Wang K, Cooney JM, Jensen DJ, et al (2009) Environmental regulation of leaf colour in red 35S:*PAP1 Arabidopsis thaliana* . New Phytol 182: 102–115. 19192188 10.1111/j.1469-8137.2008.02737.x

[24] Mishiba K, Yamasaki S, Nakatsuka T, Abe Y, Daimon H, et al (2010) Strict *de novo* methylation of the 35S enhancer sequence in gentian. PLoS One 5: e9670. 20351783 10.1371/journal.pone.0009670 PMC2843634

[25] Galis I, Schuman MC, Gase K, Hettenhausen C, Hartl M, et al. (2013) The use of VIGS technology to study plant-herbivore interactions. In: Becker A, editor. Methods in Molecular Biology - Virus-induced gene silencing: Methods and protocols. Totowa, NJ: Humana Press Inc. pp. 109–138. 10.1007/978-1-62703-278-0_9 23386299

[26] Matsuba Y, Sasaki N, Tera M, Okamura M, Abe Y, et al (2010) A novel glucosylation reaction on anthocyanins catalyzed by acyl-glucose-dependent glucosyltransferase in the petals of carnation and delphinium. Plant cell 22: 3374–3389. 20971893 10.1105/tpc.110.077487 PMC2990145

[27] Fukumoto K, Alamgir K, Yamashita Y, Mori IC, Matsuura H, et al (2013) Response of rice to insect elicitors and the role of OsJAR1 in wound and herbivory-induced JA-Ile accumulation. J Integr Plant Biol 55: 775–784. 23621526 10.1111/jipb.12057

[28] Tohge T, Nishiyama Y, Hirai MY, Yano M, Nakajima J, et al (2005) Functional genomics by integrated analysis of metabolome and transcriptome of Arabidopsis plants over-expressing an MYB transcription factor. Plant J 42: 218–235. 15807784 10.1111/j.1365-313X.2005.02371.x

[29] Pattanaik S, Kong Q, Zaitlin D, Werkman JR, Xie CH, et al (2010) Isolation and functional characterization of a floral tissue-specific R2R3 MYB regulator from tobacco. Planta 231: 1061–1076. 20157728 10.1007/s00425-010-1108-y

[30] Bai Y, Pattanaik S, Patra B, Werkman JR, Xie CH, et al (2011) Flavonoid-related basic helix-loop-helix regulators, NtAn1a and NtAn1b, of tobacco have originated from two ancestors and are functionally active. Planta 234: 363–375. 21484270 10.1007/s00425-011-1407-y

[31] Arimura G, Köpke S, Kunert M, Volpe V, David A, et al (2008) Effects of feeding *Spodoptera littoralis* on Lima bean leaves: IV. Diurnal and nocturnal damage differentially initiate plant volatile emission. Plant Physiol 146: 965–973. 18165324 10.1104/pp.107.111088 PMC2259069

[32] Staswick PE, Tiryaki I (2004) The oxylipin signal jasmonic acid is activated by an enzyme that conjugates it to isoleucine in Arabidopsis. Plant Cell 16: 2117–2127. 15258265 10.1105/tpc.104.023549 PMC519202

[33] Lulai EC, Suttle JC, Pederson SM (2008) Regulatory involvement of abscisic acid in potato tuber wound-healing. J Exp Bot 59: 1175–1186. 18356146 10.1093/jxb/ern019

[34] Boatwright JL, Pajerowska-Mukhtar K (2013) Salicylic acid: an old hormone up to new tricks. Mol Plant Pathol 14: 623–634. 23621321 10.1111/mpp.12035 PMC6638680

[35] Zhang PJ, Zheng SJ, van Loon JJ, Boland W, David A, et al (2009) Whiteflies interfere with indirect plant defense against spider mites in Lima bean. Proc Natl Acad Sci USA 106: 21202–21207. 19965373 10.1073/pnas.0907890106 PMC2795486

[36] Chung SH, Rosa C, Scully ED, Peiffer M, Tooker JF, et al (2013) Herbivore exploits orally secreted bacteria to suppress plant defenses. Proc Natl Acad Sci USA 110: 15728–15733. 24019469 10.1073/pnas.1308867110 PMC3785742

[37] Farmer EE, Ryan CA (1990) Interplant communication: airborne methyl jasmonate induces synthesis of proteinase inhibitors in plant leaves. Proc Natl Acad Sci USA 87: 7713–7716. 11607107 10.1073/pnas.87.19.7713 PMC54818

[38] Srinivasan T, Kumar KR, Kirti PB (2009) Constitutive expression of a trypsin protease inhibitor confers multiple stress tolerance in transgenic tobacco. Plant Cell Physiol 50: 541–553. 19179349 10.1093/pcp/pcp014

[39] Mori K, Goto-Yamamoto N, Kitayama M, Hashizume K (2007) Loss of anthocyanins in red-wine grape under high temperature. J Exp Bot 58: 1935–1945. 17452755 10.1093/jxb/erm055

[40] Dela G, Or E, Ovadia R, Nissim-Levi A, Weiss D, et al (2003) Changes in anthocyanin concentration and composition in ‘Jaguar’ rose flowers due to transient high-temperature conditions. Plant Sci 164: 333–340.

[41] Vaknin H, Bar-Akiva A, Ovadia R, Nissim-Levi A, Forer I, et al (2005) Active anthocyanin degradation in *Brunfelsia calycina* (yesterday-today-tomorrow) flowers. Planta 222: 19–26. 15918029 10.1007/s00425-005-1509-5

[42] Baudry A, Caboche M, Lepiniec L (2006) TT8 controls its own expression in a feedback regulation involving TTG1 and homologous MYB and bHLH factors, allowing a strong and cell-specific accumulation of flavonoids in *Arabidopsis thaliana* . Plant J 46: 768–779. 16709193 10.1111/j.1365-313X.2006.02733.x

[43] Pandey A, Misra P, Chandrashekar K, Trivedi PK (2012) Development of *AtMYB12*-expressing transgenic tobacco callus culture for production of rutin with biopesticidal potential. Plant Cell Rep 31: 1867–1876. 22733206 10.1007/s00299-012-1300-6

[44] Hettenhausen C, Baldwin IT, Wu J (2013) *Nicotiana attenuata* MPK4 suppresses a novel jasmonic acid (JA) signaling-independent defense pathway against the specialist insect *Manduca sexta*, but is not required for the resistance to the generalist *Spodoptera littoralis* . New Phytol 199: 787–799. 23672856 10.1111/nph.12312 PMC4996321

