# Supplementary figures and images for: Overexpression of the PAP1 Transcription Factor Reveals a Complex Regulation of Flavonoid and Phenylpropanoid Metabolism in Nicotiana tabacum Plants Attacked by Spodoptera litura
Source: PLoS One. 2014 Sep 30;9(9):e108849. doi: 10.1371/journal.pone.0108849 (PMC4182574; doi:10.1371/journal.pone.0108849)

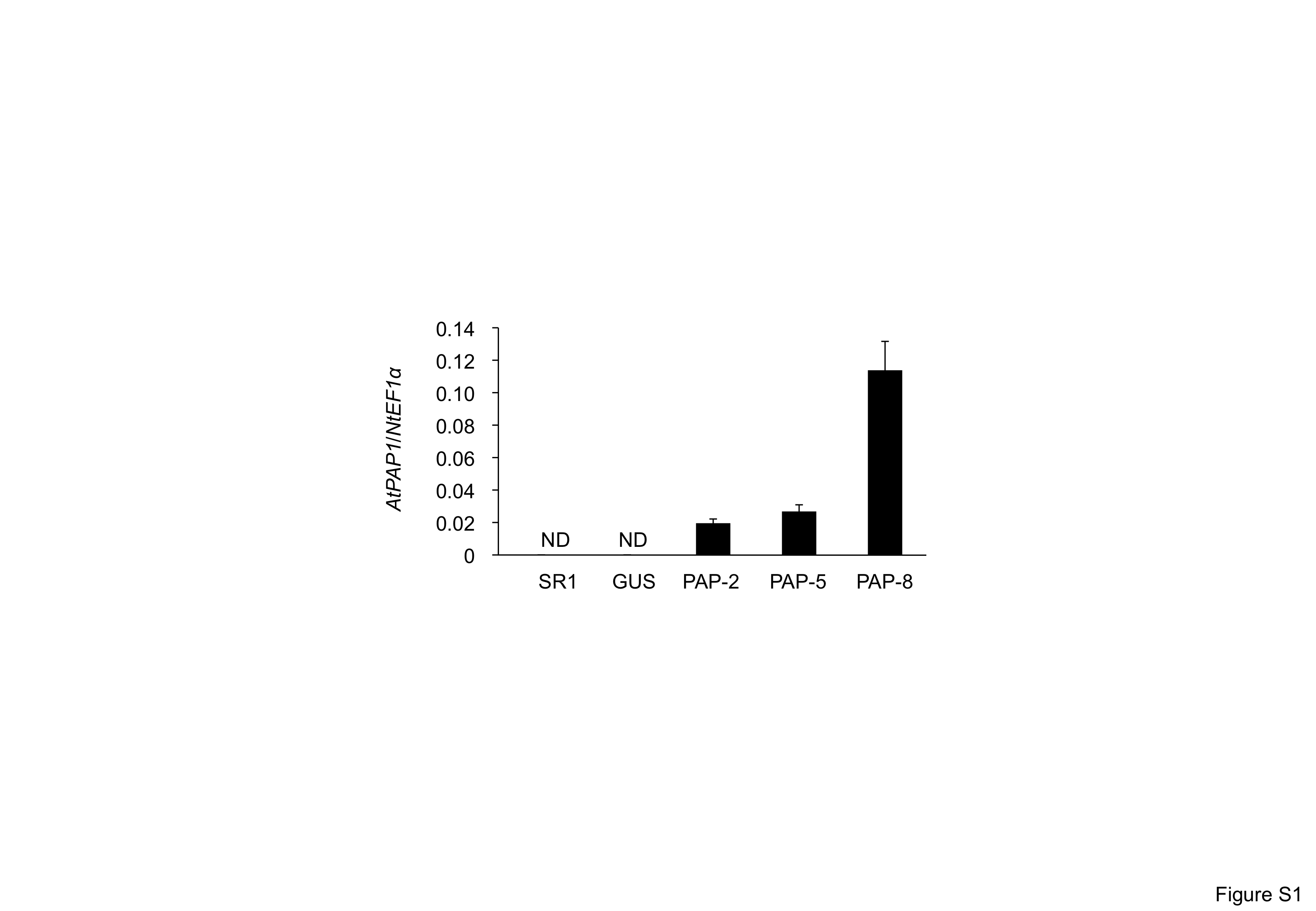

Supplement: Figure S1 — Relative mRNA levels of PAP1 in leaves of wild-type (WT), GUS and PAP1 lines. Transcript levels of genes were normalized by those of NtEF1α. Data are shown as the mean+standard errors (n = 5). ND, not detected. (TIF) [file pone.0108849.s001.tif]

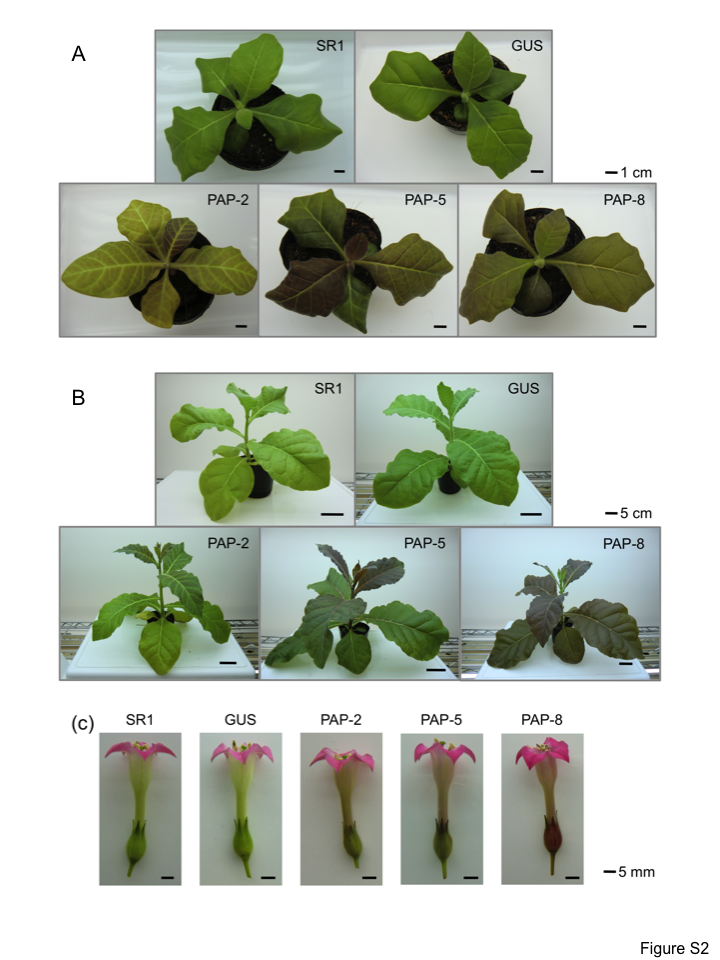

Supplement: Figure S2 — Morphology of PAP1 lines. A, 4-week-old plants; B, 8-week-old plants; C, flowers. (TIF) [file pone.0108849.s002.tif]

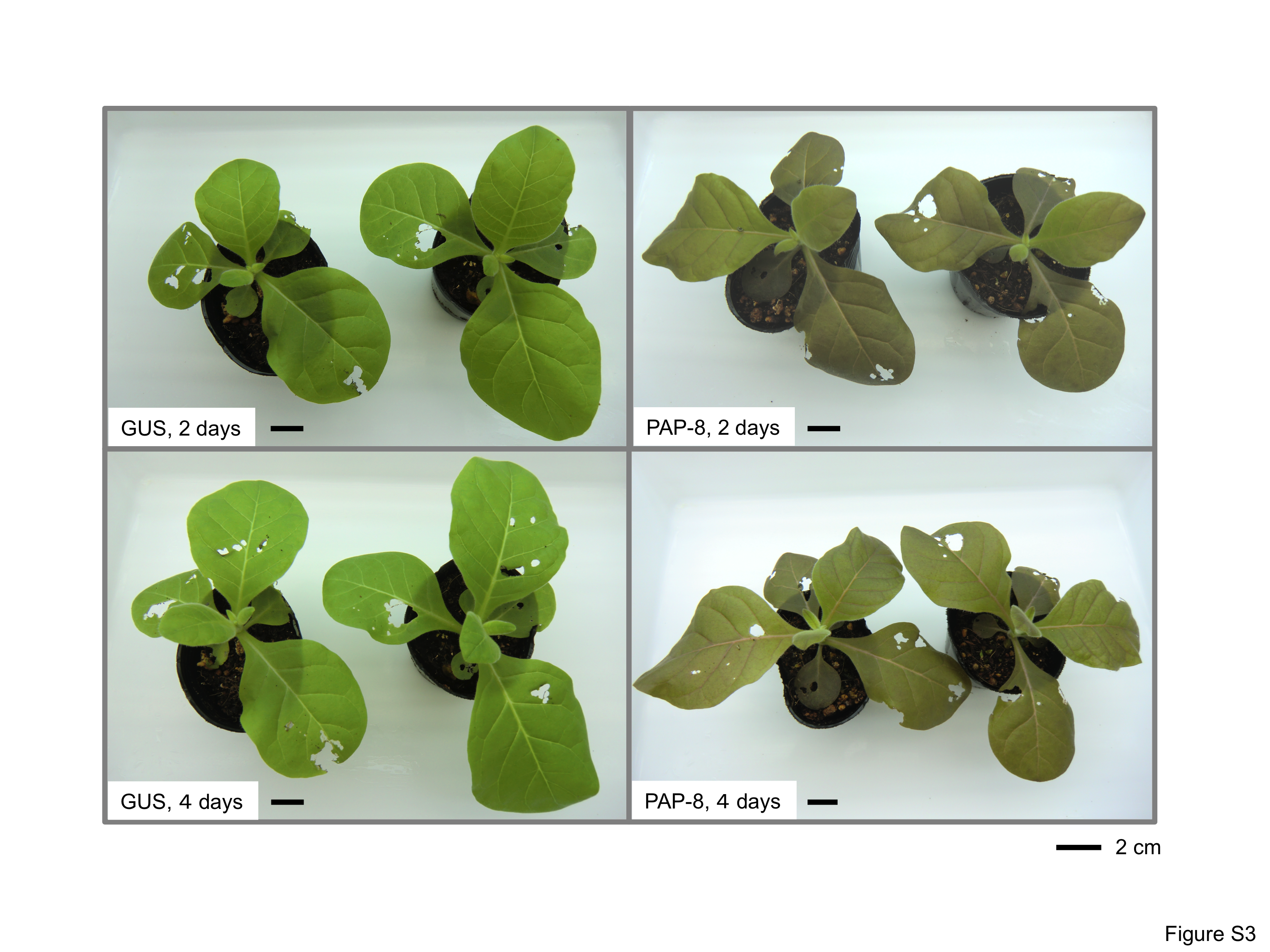

Supplement: Figure S3 — GUS and PAP-8 plants infested with Spodoptera litura for 2 days (A) and 4 days (B). (TIF) [file pone.0108849.s003.tif]

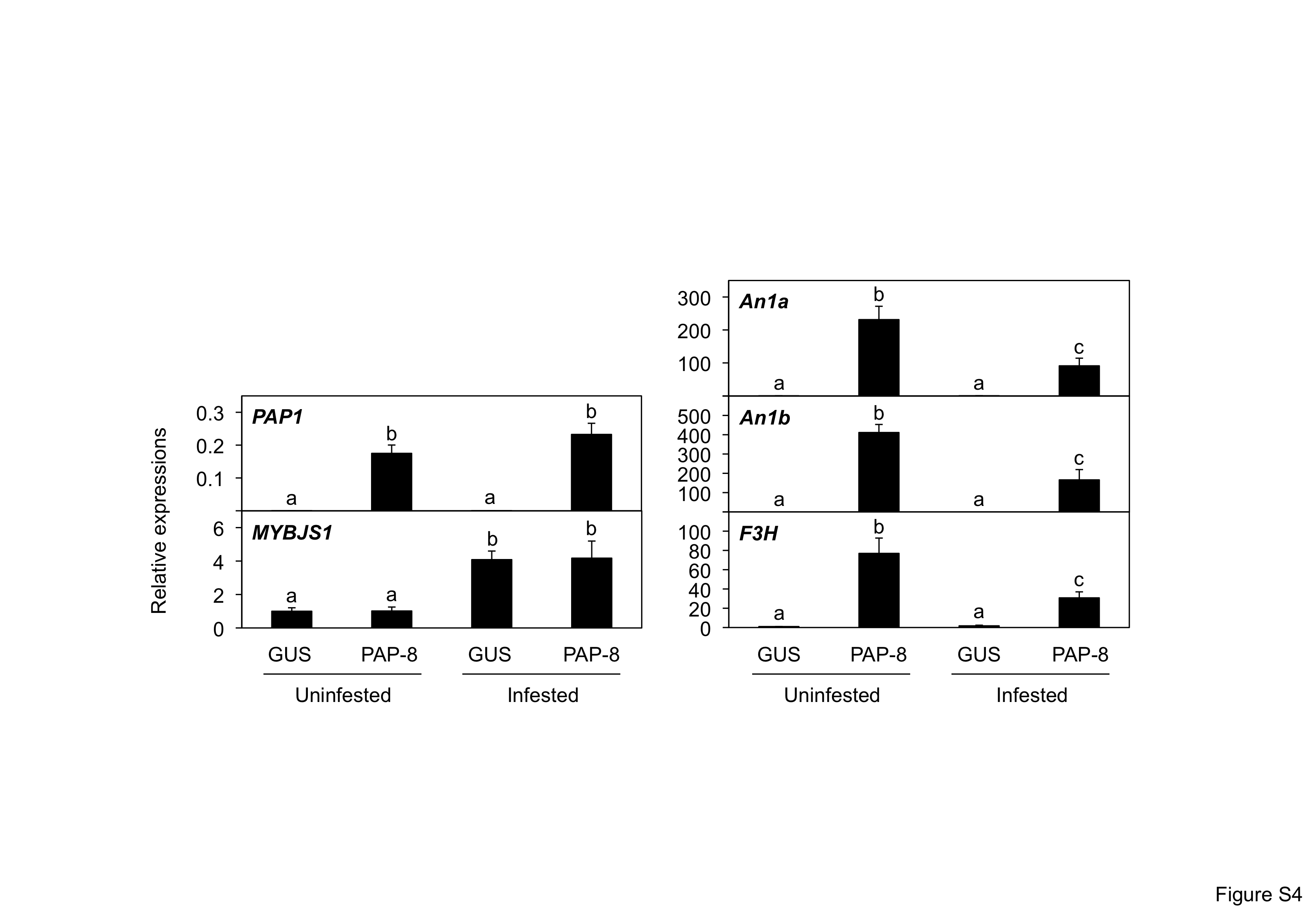

Supplement: Figure S4 — Expression of genes in GUS and PAP-8 leaves infested with Spodoptera litura for 4 days. Relative transcript levels of genes were determined in leaves of GUS control and PAP1 lines (PAP-8) not infested or infested with S. litura for 4 days. Transcript levels of genes were normalized by comparing them to those of NtEF1α. Data are shown as the mean+standard errors (n = 4–5). Means followed by different small letters are significantly different (P<0.05). (TIF) [file pone.0108849.s004.tif]
